# Trust in the referring physician reduces anxiety in an integrated community-to-hospital care system

**DOI:** 10.1186/s13584-020-00365-6

**Published:** 2020-05-11

**Authors:** Moshe Y. Flugelman, Ronen Jaffe, Gil Luria, Dana Yagil

**Affiliations:** 1 Department of Cardiovascular Medicine, Lady Davis Carmel Medical Center, 7 Michal Street, 34632 Haifa, Israel; 2 Rappaport Faculty of Medicine, Technion Israel Institute of Technology, Haifa, Israel; 3 Department of Human Services, University of Haifa, Haifa, Israel

**Keywords:** Continuity of care, Trust, Anxiety, Hospitalization

## Abstract

**Background:**

Continuity of care between the community and hospital is considered of prime importance for quality of care and patient satisfaction, and for trust in the medical system. In a unique model of continuity of care, cardiologists at our hospital serve as primary, community-based cardiologists one day a week. They refer patients from the community to our hospital for interventional procedures such as coronary angiography and angioplasty. We examined the hypotheses that patient anxiety during hospital-based coronary angiography is lower when a patient trusts the referring cardiologist and when the performing cardiologist also treated him/her in the community.

**Methods:**

We administered questionnaires to 64 patients in our cardiology department within 90 min of completion of coronary angiography. The questions assessed anxiety, trust in the medical system and trust in the referring physician. Data were also collected regarding patients’ demographic variables, the number of visits to the referring physician, and whether the physician who performed the coronary angiography was the physician who referred the patient to the hospital.

**Results:**

Mean levels (on 7-point Likert scales) were 2.1, 5.6 and 6.7 for patient anxiety, trust in the medical system and trust in the referring physician, respectively. Multivariate regression analysis showed that trust in the referring physician was significantly and negatively correlated with anxiety level. The number of visits to referring physicians, patients’ demographic characteristics and whether the physician who performed the angiography was the same physician who referred the patient from the community were not found to be associated with patient anxiety.

**Conclusion:**

In this study, trusting the referring physician was associated with lower anxiety among patients who underwent coronary angiography. This trust seemed to have more positive impact than did previous contact with the physician who performed the procedure.

## Background

Continuity of care has been identified as a major component of high-quality care and of patients’ satisfaction [1–4]. Continuity of care between community and hospital settings is particularly challenging, as it involves bilateral transfer of information and coordination over time. Fragmentation of care can reduce patients’ trust in the medical system and in physicians, and increase anxiety prior to interventions in an unfamiliar setting such as hospitals [5–10]. Moreover, a number of models of encounters between patients and previously unknown physicians have highlighted the impact of trust between the physician and the patient on reducing anxiety [11, 12].

Over the last three decades, we have employed a unique model for continuity of care for cardiology [5, 13]. Hospital-based cardiologists serve as primary, community-based cardiologists one day a week. They refer their patients in the community to our hospital for interventional procedures such as coronary angiography and angioplasty. Indications for coronary angiography are re-evaluated in the hospital, and if confirmed, patients undergo the procedure. If the referring cardiologist is an interventional cardiologist, we try to arrange that this cardiologist will perform the angiography and angioplasty for the patient he/she referred from the community.

Based on our continuity of care model, we examined the hypotheses that patient anxiety during hospital-based coronary angiography is lower when a patient trusts the referring cardiologist, and when the performing cardiologist also treated him/her in the community. To test our hypotheses, we interviewed patients who underwent coronary angiography and angioplasty, regarding their level of anxiety, their trust in the medical system, and their trust in the physician who referred them. We compared responses between patients according to whether the cardiologist who referred them also performed the coronary angiography and angioplasty.

## Methods

### Participants

The sample consisted of 64 patients in the Department of Cardiovascular Medicine who had undergone coronary angiography and angioplasty. Fifty-six (88%) of the patients reported the number of visits they had in the community with the physician who referred them to the procedure; 29% reported one visit, 36% two visits, 21% three visits and 14% reported four visits or more.

Inclusion criteria were referral to elective coronary angiography, and the capability and willingness to participate in the study. Patients with acute coronary syndromes were excluded from the study.

### Procedure

The institutional review board of Lady Davis Carmel Medical Center waived the need for approval of this study. Hospitalized patients who were after coronary angiography were asked to participate in the study. There were no refusals to participate. Patients with unstable medical conditions were not approached regarding participation. Interviewers were conducted by senior nursing students who received particular training for this study and who were introduced to the nursing staff at the catheterization inpatient unit. Since questionnaires were in Hebrew, the interviewers translated the questionnaires to patients who needed translation or clarification in additional languages (Arabic and Russian). As the interviewers speak Hebrew and at least one additional language, the use of an external translator was infrequent. Questionnaires were administered to respondents by the interviewers within 90 min of completion of coronary angiography and angioplasty.

### Measures

*State anxiety* was measured with 13-items from the Trait Anxiety Inventory (STAI) [14], which was previously used to measure cardiac patients’ anxiety [15]. An example item is: “I feel tense”. Participants were asked to indicate on a 7-point Likert scale (1 = “not at all”; 7 = “very much”) their feelings at the moment. Cronbach’s alpha reliability was 0.87.

*Trust in the medical system* was measured with a 4-item scale [16]. An example item is: “To what extant do you believe that the medical system puts your medical needs above all other matters?” Participants were asked to respond according to a 7-point Likert scale (1 = “not at all”; 7 = “very much”). Cronbach’s alpha reliability was 0.66.

*Trust in the physician* who referred the patient to catheterization was measured with a 5-item scale [16]. An example item is: “To what extent do you believe your doctor chose the best medical treatment for you?” Participants were asked to respond according to a 7-point Likert scale (1 = “not at all”; 7 = “very much”). Cronbach’s alpha reliability was 0.63.

*Demographic characteristics and medical encounter details* were accessed from the patients: age, education, the number of visits to the physician who referred them to catheterization, and whether the physician who referred them also performed the procedure.

*Self-efficacy*, meaning that a person feels capable of accomplishing tasks [17], was also assessed using our questionnaire. Scoring was according to a 7-point Likert scale (1 = “not at all”; 7 = “a very high level of self-efficacy”).

### Statistical analysis

To predict patients’ anxiety, we conducted a multiple linear regression analysis. Independent variables were entered in three phases to evaluate their distinct contributions. Demographic variables (i.e., gender, age and education) were entered in the first phase. The number of visits to the physician and whether the physician who referred them to catheterization also performed the procedure were entered in the second phase. Trust in the medical system and trust in the physician who referred the patient were entered in the third phase.

To examine the association of the familiarity of the referring physician with patient anxiety we conducted a one-way analysis of variance. We used SPSS 21 version for statistical analysis.

## Results

Fifty-two (81%) of the patients reported the identity of the physician who referred them to the procedure. Seventeen (27%) reported that the physician who referred them was the same physician who performed the procedure, 24 (38%) reported being referred by a physician who works in the hospital where the procedure was performed and 11 (17%) were referred by another physician. Means and standard deviations of the variables examined, and correlations between them are shown in Table 1. The mean age of the respondents was 66.8 years (*SD* = 10.8), their mean years of education was 13.1 (*SD* = 2.75); 45 (70%) were men.

**Table 1:** Means, standard deviations and correlations of the variables investigated

	Mean (standard deviation)	1	2	3	4	5
1.Anxiety	2. 0 (.73)					
2.Trust in the medical system	5.58 (1.41)	−.28*				
3.Trust in the physician	6.66 (.60)	−.59**	.31*			
4.Number of visits		.25	.09	.08		
5.Age	66.84 (10.09)	−.03	−.04	.06	−.04	
6.Education	13.06 (2.95)	−.05	−.21	.08	−.02	−.06

*p < .05; **p < .01

Mean levels (on 7-point Likert scales) for patient anxiety, trust in the medical system and trust in the physician were 2.1, 5.6 and 6.7, respectively. Significant inverse correlations were observed between anxiety and trust in the physician, and between anxiety and trust in the system. Scores on self-efficacy did not differ between the groups.

We conducted a multiple linear regression analysis of the variables that were associated with anxiety (Table 2), using the three stages detailed in the statistical methods section. The results of this analysis indicated that trust in the referring physician was negatively related to anxiety, beyond the effect of the other variables.

**Table 2:** Regression analysis of various factors (demographic factors, medical encounter details, and trust in the system and in the physician) on patient anxiety

Variable 3	Phase 1			Phase 2			Phase 3		
	*B*	*SE* (*B*)	Β	*B*	*SE* (*B*)	β	*B*	*SE* (*B*)	β
Gender	.28	.21	.20	.27	.22	.20	.28	.20	.21
Age	.01	.01	.20	.01	.01	.18	.01	.01	.19
Education	.00	.03	.03	.00	.00	.03	.00	.03	.03
Number of visits				.02	.06	.05	.05	.06	.13
Referring physician performed/did not perform the procedure				.09	.13	.11	.07	.12	.09
Trust in the medical system							−.10	.07	−.23
Trust in the physician							−.37	.18	−.31*
*R*^2^			.07			.09			.26
*F*			.99			.69			1.75

*p < .05

Anxiety level was similar between the patient groups, according to the patient’s relation with the performing physician. For patients for whom the physician who referred them to angiography was the same physician who performed the procedure, the mean value was 2.09 ± 0.9 (Table 3). For patients who were referred by a physician who works in the hospital where the procedure was performed, the mean value was 2.19 ± 0.7. For patients who were referred by another physician, the mean value was 1.92 ± 0.6 (Table 3).

**Table 3:** Patients’ attitudes toward physicians and the medical system, according to familiarity with the catheterizing physician 1.92 ± 0.6

Variable	Catheterizing physician	Number of patients	Mean ± SD	*p* Value
Anxiety	Referring	17	2.09 ± 0.9	NS
	Non referring	24	1.92 ± 0.6	
	Same department	11	2.19 ± 0.7	
Satisfaction with the physician	Referring	17	6.4 ± 0.7	NS
	Non referring	22	6.4 ± 0.9	
	Same department	10	6.0 ± 0.8	
Trust in the physician	Referring	17	6.6 ± 0.8	NS
	Non referring	24	6.8 ± 0.5	
	Same department	11	6.6 ± 0.6	
Trust in the system	Referring	17	5.14 ± 1.7	NS
	Non referring	24	5.66 ± 1.5	
	Same department	11	5.63 ± .83	
Self-efficacy	Referring	16	6.4 ± 1.0	NS
	Non referring	24	6.8 ± 0.5	
	Same department	11	6.8 ± 0.4	

## Discussion

Our findings supported our hypothesis of an inverse association of patient anxiety with trust in the referring cardiologist. However, the findings do not support our hypothesis of higher trust and lower anxiety among patients for whom the cardiologist who performed the intervention had referred him/her. Rather, trust in the referring physician was a stronger determinant of anxiety than was the prior relationship of the patient with the physician who performed the angiography. Trust in the physician was also a stronger determinant of anxiety than any of the demographic characteristics examined. Our findings suggest that the “holy grail” of reducing patient anxiety during hospitalization does not entail being treated by the same physician as in the community, but rather, trusting the referring physician regardless as to whether s/he performed the procedure in the hospital.

The components that instill trust are detailed by Bachrach et al. in the seven dimensions’ model of continuity of care [9]. Hennen described four dimensions of continuity of care in family practice: chronological, geographical, interdisciplinary and interpersonal [18]. In agreement, our work suggests that trust can be generated despite differences in geography and in disciplines, and despite treatment by unfamiliar physicians.

Findings similar to ours were described by Hinnen et al., who showed an association between lower trust in one’s physician and higher rates of distress among patients with cancer who had attachment anxiety [19]. Moreover, the inverse association between anxiety and empathic response from a physician was shown in a study on hospital admissions. There, the frequency of empathic responses by the physicians was associated with trust and with the perception of being cared by the physician [20].

Contrary to our expectations, our data did not support a substantial role of relational continuity in the level of anxiety during hospitalization. Rather, our data showed low anxiety overall. We presume that the high quality of care of our system, and the high level of trust that our patients attribute to their referring physicians may help overcome gaps between the community and hospital, and thus reduce patients’ anxiety. The literature is inconclusive regarding associations of healthcare continuity with patients’ satisfaction and with quality of healthcare. Accordingly, one systematic review found a variable effect of continuity on patients’ satisfaction [21], while a more recent meta-analysis showed reduced mortality with continuity of care [22].

There are several limitations to our study. These include the relatively small sample size and the timing of the interviews, which were conducted after completion of a medical procedure. While the latter may have affected our findings, the administration of diazepam as pre-procedural medication to all the patients precluded conducting the interviews prior to the procedure. The high levels of patient satisfaction may hinder our capacity to detect differences between patients according to familiarity with hospital physicians, and Cronbach’s alpha reliability was of borderline level in this study. Based on the abovementioned issues, our findings should be regarded as preliminary, and a larger scale study should be considered.

## Conclusions

This study showed that trust in the referring physician is a strong determinant of lower anxiety during procedures in the hospital. This trust was independent of the familiarity of the patient with the physician who performed the procedure. Our findings merit additional studies with a larger number of patients to examine the association between patients’ trust and anxiety in various settings.

## Data Availability

Data are available upon request.
